# Study to prospectively evaluate reamed intramedually nails in patients with tibial fractures (S.P.R.I.N.T.): Study rationale and design

**DOI:** 10.1186/1471-2474-9-91

**Published:** 2008-06-23

**Authors:** The SPRINT Investigators

**Affiliations:** 1 SPRINT Methods Center, Department of Clinical Epidemiology and Biostatistics, 1200 Main Street West, Room 2C9 Hamilton, Ontario, Canada

## Abstract

**Background:**

Surgeons agree on the benefits of operative treatment of tibial fractures – the most common of long bone fractures – with an intramedullary rod or nail. Rates of re-operation remain high – between 23% and 60% in prior trials – and the two alternative nailing approaches, reamed or non-reamed, each have a compelling biological rationale and strong proponents, resulting in ongoing controversy regarding which is better.

**Methods/Design:**

The objective of this trial was to assess the impact of reamed versus non-reamed intramedullary nailing on rates of re-operation in patients with open and closed fractures of the tibial shaft. The study to prospectively evaluate reamed intramedullary nails in tibial fractures (S.P.R.I.N.T) was a multi-center, randomized trial including 29 clinical sites in Canada, the United States and the Netherlands which enrolled 1200 skeletally mature patients with open (Gustilo Types I-IIIB) or closed (Tscherne Types 0–3) fractures of the tibial shaft amenable to surgical treatment with an intramedullary nail. Patients received a statically locked intramedullary nail with either reamed or non-reamed insertion. The first strategy involved fixation of the fracture with an intramedullary nail following reaming to enlarge the intramedullary canal (Reamed Group). The second treatment strategy involved fixation of the fracture with an intramedullary nail without prior reaming of the intramedullary canal (Non-Reamed Group). Patients, outcome assessors, and data analysts were blinded to treatment allocation. Peri-operative care was standardized, and re-operations before 6 months were proscribed. Patients were followed at discharge, 2 weeks post-discharge, and at 6 weeks, 3, 6, 9, and 12 months post surgery. A committee, blinded to allocation, adjudicated all outcomes.

**Discussion:**

The primary outcome was re-operation to promote healing, treat infection, or preserve the limb (fasciotomy for compartment syndrome after nailing). The primary outcome was a composite comprising the following re-operations: bone grafts, implant exchanges, and dynamizations, in patients with fracture gaps less than 1 cm post intramedullary nail insertion. Infections and fasciotomies were considered events irrespective of the fracture gap. We planned *a priori *to conduct a subgroup analysis of outcomes in patients with open and closed fractures. S.P.R.I.N.T is the largest collaborative trial evaluating alternative orthopaedic surgical interventions in patients with tibial shaft fractures. The methodological rigor will set new benchmarks for future trials in the field and its results will have important impact on patient care. The S.P.R.I.N.T trial was registered [ID NCT00038129] and received research ethics approval (REB#99-077).

## Background

The S.P.R.I.N.T trial protocol was developed as a multi-center, blinded randomized controlled trial (RCT) to compare alternative intramedullary techniques in 900 patients with closed and open tibial shaft fractures. The trial received joint funding from the Canadian Institutes of Health Research and the National Institutes of Health. This article provides the rationale and design of the original S.P.R.I.N.T protocol. We also provide a detailed report of the major protocol changes made during the study execution. Specifically, we present changes made in response to the first interim analysis including a revision of our sample size estimates and a refinement of the primary composite outcome.

### Magnitude of the problem

Fractures of long bones constitute the majority of emergency operating room procedures in most trauma centres. Of these long bone injuries, tibial fractures are the most common. The National Center for Health Statistics reports an annual incidence of 492,000 fractures of the tibia and fibula per year in the United States [1]. Patients with tibial fractures remain in hospital for a total of 569,000 hospital days and incur 825,000 physician visits per year in the United States [1].

Tibial fractures are prone to complications [2-5]. The lack of a circumferential soft tissue envelope around the bone makes the bone ends more likely to fail to unite (nonunion). Approximately 50,000 North Americans suffer each year from these nonunion complications [6]. Nonunions may require secondary operations to promote fracture healing. In addition to the risk of general anesthesia and early post-operative venous thromboembolism complications [7-9], patients who require re-operation face additional rehabilitation and time off work. Furthermore, re-operations result in substantial resource consumption and indirect costs due to decreased productivity. Management strategies to best minimize these frequent complications and resulting re-operations have proved controversial.

### Historical management of tibial fractures

Over the last 20 years, surgeons have used four management approaches for tibial fractures: intramedullary nail fixation [10-12], plate fixation [13], external fixation [14] and casting or functional bracing [15-17]. While it may be preferable to cast a closed tibial shaft fracture, most surgeons agree that in certain unstable fracture patterns, casts will not maintain adequate fracture alignment [18]. In recent years, surgeons have moved away from plates and external fixators in favour of intramedullary nails in the operative treatment of both closed and open tibial fractures [19]. To clarify this issue, we conducted a meta-analysis examining evidence regarding the treatment of open tibial fractures. We identified randomized trials (RCTs) comparing plates with external fixators [20] and intramedullary nails with external fixators [21-25]. The pooled estimate from 5 studies (n = 396 patients) that compared external fixators to non-reamed intramedullary nails showed a large reduction in the risk of re-operation with non-reamed nails, and an associated narrow confidence interval (relative risk 0.51, 95% CI 0.37–0.69) [21-25]; therefore, we can be confident that intramedullary nailing will reduce the rate of re-operation by over one third. Although most surgeons agree that intramedullary nails are the preferred treatment, the choice of reamed versus non-reamed nail insertion remains controversial.

### Rationale for the two dominant approaches

Surgeons currently favor the use of interlocking intramedullary nails to reduce both nonunion and infection rates; however, they do not agree on the optimal approach to nailing. Reaming of the medullary canal, with placement of a large nail to ensure optimal biomechanical stability while promoting healing, and the use of a non-reamed nail, to maintain blood flow to the cortical bone to promote healing, both have strong rationale and ardent advocates.

#### Reamed Intramedullary Nails

Research supporting the use of reamed intramedullary nails has focused on the increased blood flow to soft tissues and the superior biomechanical stability of reamed nails. For example, Schemitsch et al have shown in a sheep tibial fracture model that reaming prior to nail insertion significantly increases muscle and surrounding soft tissue blood flow in comparison to unreamed nails. This increase persists for up to 6 weeks [26]. Utvag et al confirmed these findings in a rat femoral fracture model [27]. Increased blood flow to soft tissue may also improve cortical blood flow: Grundnes and colleagues have demonstrated 5-fold increases in cortical blood flow following reamed nailing of rat femurs when compared to the control [28]. In addition to possible advantages from increased blood flow, investigators have documented the biomechanical superiority of large diameter nails versus smaller diameter nails [29,30].

#### Non-Reamed Intramedullary Nails

Massive destruction of the endosteal blood supply (up to 70%) following intramedullary reaming has been independently reported by Rhinelander and Olerud [31-33]. In a canine tibial fracture model, Klein et al. have shown that non-reamed nailing also disturbs cortical circulation [34]. Using a similar model, Hupel et al. have demonstrated that a non-reamed intramedullary nail allows superior cortical revascularization at 11 weeks when compared to a tight-fitting nail [35]. Schemitsch has shown that significant increases in cortical bone porosity are associated with reamed intramedullary nails [36].

In summary, experimental data suggest that reamed nails offer greater biomechanical stability and increased soft tissue blood flow, while non-reamed nails preserve blood flow to the bone. While both arguments are persuasive, evidence of the impacts of these biologic alterations on outcomes that are important to patients would be more compelling data in guiding clinical practice.

### Reamed and non-reamed nails: effect on important outcomes

Both nonunion of the bone [37-40] and infection at the implant-bone interface [41,42] may necessitate a second operation to promote fracture healing. Because of the risks and costs to the patient, and the costs to the health care system, reoperation represents an important outcome from both individual and societal points of view. Our meta-analysis of RCTs [43] suggested that reamed intramedullary nailing of lower extremity femoral and tibial bone fractures results in significantly fewer nonunions than non-reamed nailing [44-52] (pooled relative risk 0.33, 95% CI 0.16 to 0.68). Evaluation of fracture subgroups further suggested a large treatment effect favoring reamed nails in femoral fractures (relative risk: 0.24, 95% CI, 0.07–0.82), but a less persuasive effect in tibial fractures (relative risk 0.44, 95%CI 0.21–0.93) [43].

Because the biology of open tibial fractures is different from that of closed fractures, the relative impact of reamed and non-reamed nailing may differ in these sub-populations. The significant soft tissue damage and stripping of the periosteum from the cortical bone, which is common in open fractures, has the potential to compromise blood supply to that region [53]; therefore, the preservation of endosteal, or intramedullary, blood supply may be more important. Opponents of reamed nails believe that the disruption of endosteal blood supply, as a result of intramedullary reaming, increases the risk of nonunion for the fracture.

To clarify this issue, we conducted a second meta-analysis examining evidence regarding the treatment of open tibial fractures [54]. We identified two small RCTs that compared reamed and non-reamed intramedullary nails. The small sample sizes (total n = 132) in the two studies that have compared reamed nails [44,50] is reflected in the wide confidence intervals around the trend in favor of reamed nails in the risk of re-operation (0.75, 95% CI, 0.43–1.32) in comparison to non-reamed nails. The trends in favor of reamed nails in open tibial fractures are consistent with findings of our prior meta-analysis.

### Is the answer in for tibial fractures?

Despite the results of these two meta-analyses, uncertainty about the optimal treatment for tibial fractures remains. Reasons for this uncertainty ensue:

1. The tibia differs biologically from the femur because it does not have a circumferential soft tissue envelope that partly provides the blood supply to the bone. While the intact soft tissue envelope around the femur is adequate to maintain blood supply to the bone and promote fracture healing following intramedullary reaming, this may not be the case for tibial shaft fractures. Thus, the biological case for reaming is weaker in tibial fractures.

2. While the evidence from our first meta-analysis strongly favours the use of reamed intramedullary nails in the treatment of femoral fractures (relative risk: 0.24, 95% CI, 0.07–0.82), the effects of reamed nails in tibial fractures are not as persuasive (relative risk 0.44, 95%CI 0.21–0.93) [43].

3. Current opinions in the treatment of tibial shaft fractures among orthopaedic traumatologists remain divergent. We previously surveyed a 20% random sample of members of the Canadian Orthopaedic Association. Of the 60 respondents who treat tibial fractures, 35 (58%) indicated that they thought reamed nails were superior and 25 indicated (42%) that they thought non-reamed nails were the same or better than reamed nails. For open tibial fractures: 31 (52%) believed non-reamed nails were superior whereas 29 (48%) believed reamed nails were the same or better. The results demonstrate a lack of predominant nail preference for both open and closed fractures. Our findings were supported in a large international survey of surgeons (Table 1).

**Table 1:** Implant preference among surgeons (N = 444)

Type of Fracture	Type of Implant (%)			
	External Fixator	Plate	IM Nail (Reamed)	IM Nail (Non-Reamed)

**CLOSED FRACTURES**				
Closed Fractures (Low Energy) *	0.5	3.2	76.0	20.3
Closed Fractures (High Energy)	1.8+	2.1	60.4+	35.6+
Closed Fractures with Compartment Syndrome	12.2+	7.4+	34.9+	45.5+
**OPEN FRACTURES**				
Grade I Open Fractures	3.4	1.1	54.5	41.0
Grade II Open Fractures	11.1#	0.8	46.3#	41.8
Grade IIIa Open Fractures	30.6#	1.1	28.8#	39.6
Grade IIIb Open Fractures	50.5#	1.1	13.6#	34.8

* 0.8% respondents treated all injuries by non-operative methods. IM = intramedullary

+ significant difference when compared to responses for closed fractures (low energy)

# significant difference when compared to responses for Grade I open fractures (p < 0.01)

From: Bhandari M, Guyatt GH, Swiontkowski MF, Tornetta P 3rd, Hanson B, Weaver B, Sprague S, Schemitsch EH. Surgeons' preferences for the operative treatment of fractures of the tibial shaft. An international survey. J Bone Joint Surg Am. 2001;83-A:1746-52

Only a large RCT would resolve the remaining, legitimate, scientific uncertainty, and the continuing controversy in the clinical community.

## Methods/Design

The primary objective was to assess the impact of reamed and non-reamed intramedullary nailing on rates of re-operation (including nail exchange, bone grafts, dynamization, and fasciotomy) in patients with post-operative fracture gaps less than 1 cm following intramedullary nailing and re-operation for deep infection irrespective of the post-operative fracture gap.

The secondary objective was to assess the impact of reamed and nonreamed intramedullary nailing on functional status (Short Form-36 (SF-36), Short Musculoskeletal Functional Assessment (SMFA), Health Utilities Index (HUI), Tibia Knee Pain Questionnaire) and return to normal activities.

### Trial design

We initially proposed to enroll 900 patients in a prospective, RCT in which surgeons managed individuals who sustained a fracture of the tibia by one of two strategies. The first strategy involved fixation of the fracture with an intramedullary nail following reaming of the intramedullary canal (Reamed Group). The second treatment strategy involved fixation of the fracture with a smaller intramedullary nail without prior reaming of the intramedullary canal (Non-Reamed Group). Patients enrolled in the trial received post-operative care according to the same standards and protocols. We monitored critical aspects of pre-operative and post-operative care and provided immediate feedback to the participating surgeons when any important deviation from protocol occurred. Patients, outcome assessors, and data analysts were blinded to treatment allocation. We monitored re-operation rates at, discharge, 2 weeks post discharge, 6 weeks, 3, 6, 9, and 12 months. In patients with prolonged hospital stays, post-operative follow up forms were completed at their scheduled times and the 2 week post discharge form was completed as originally described (two weeks after the discharge date regardless of hospital stay). We received research ethics approval (REB#99-077) for this study.

### Trial interventions

#### Reamed nail insertion

Reaming was conducted over the guide wire with cannulated power reamers. The operating surgeon chose the reamer. To avoid inconsistencies in the degree of reaming, surgeons adhered to the following protocol: 1) Surgeons reamed the intramedullary canal until the first detection of "cortical chatter" (i.e. the reamer just begins to contact the cortical bone of the tibia). 2) The size of the nail (diameter) corresponded to the point of "cortical chatter" (if chatter occurred with a 11 mm reamer, then the nail size is 11 mm. 3) Following the appearance of "cortical chatter", surgeons reamed 1–1.5 mm larger than the chosen nail's diameter to facilitate its insertion. The chosen nail, which was as long as possible without distracting the fracture by impinging on dense distal metaphyseal bone or protruding above the cortex at the insertion site, was inserted with the appropriate instruments. Distraction of a tibial shaft fracture interferes with healing, so surgeons employed all strategies for achieving cortical contact (up to 10 mm shortening acceptable to achieve contact of fracture ends). The choice of intramedullary nail (i.e. company) and material (titanium or stainless steel) was at the discretion of the operating surgeon.

#### Non-reamed nail insertion

Surgeons inserted non-reamed nails across the fracture site with great attention to the prevention of over-distraction. The goal was to achieve cortical contact of the fracture ends. An upper diameter limit of 10 mm was employed for non-reamed nails. Surgeons were instructed that in principle, the nail should be at least 2 mm less than the diameter at the isthmus of the tibia on anteroposterior and lateral radiographs.

#### Interlocking screws

All fractures were interlocked, both proximally and distally. Surgeons used at least one proximal locking screw and one distal locking screw. The number of screws, one, two, or three was left to the discretion of the surgeon.

#### Peri-operative treatment common to both groups

To ensure similar peri-operative regimens, participating centres standardized key aspects of pre- and post-operative care.

In Closed Fractures: 1) Pre-operative antibiotic administration was continued for 24 hours post-operatively (specific antibiotic regimens at the discretion of the operating surgeon: gram positive coverage). 2) Cortical contact of the fracture ends guided weight bearing. If cortical contact was achieved, patients were allowed to weight bear as tolerated. However, when cortical contact was not achieved, patients are allowed to partially weight bear on the affected limb until a definitive procedure to achieve contact was performed. 3) Dynamization, a technique in which the interlocking screws are removed distally to allow compression at the fracture site, was allowed prior to 6 months only if the fracture is distracted following nail insertion. 4) Participating surgeons did not offer stimulation modalities to promote bone growth including ultrasound and electrical stimulation during the one-year of follow-up.

In Open Fractures: 1) Pre-operative intravenous antibiotic administration included a cephalosporin and an aminoglycoside which were continued for 72 hours post-operatively (specific antibiotics used at the discretion of the attending surgeon. The recommended guidelines included: cephalosporin (ancef) I.V. for Grade I-II injuries, ancef I.V. and aminoglycoside (gentamycin) I.V. for Grade III injuries, and ancef I.V., gentamycin I.V. and penicillin for gross contaminated injuries). 2) Copious irrigation and debridement of soft tissues and contaminated bone was repeated as necessary, 3) Delayed wound closure, split thickness skin grafting, or muscle flaps (for grade IIIB only) occurred by 7 days following the initial surgery. 4) Weight bearing, dynamization, and the use of stimulation modalities as per closed fractures.

### Randomization

We stratified patients by centre and according to whether they had sustained a closed or open fracture of the tibia. We randomized in blocks; clinical centres were unaware of the block size. We defined the patient as the unit of randomization. Therefore, single patients with bilateral tibial shaft fractures were randomized to one treatment alternative only. Both fractures were treated with either reamed or non-reamed nailing. We anticipated that randomization would lead to approximately the same number of such patients in the two groups. We considered single or bilateral fractures as an additional stratification variable, but decided that the risk of empty cells was too great.

The research coordinator or resident/fellow ensured that the patient met all eligibility and, as close to surgery as possible, called the 24-hour telephone computer randomization system (toll free number) at the methods centre. The research coordinator or resident/fellow entered their unique hospital site code, patient hospital identification number, and the type of fracture (open, closed, or both). Following this procedure, the computer then provided the caller with a treatment allocation (reamed or non-reamed intramedullary nail). This procedure guaranteed concealed randomization. Also, the system does not allow patients to be randomized more than once.

**Figure 1:**
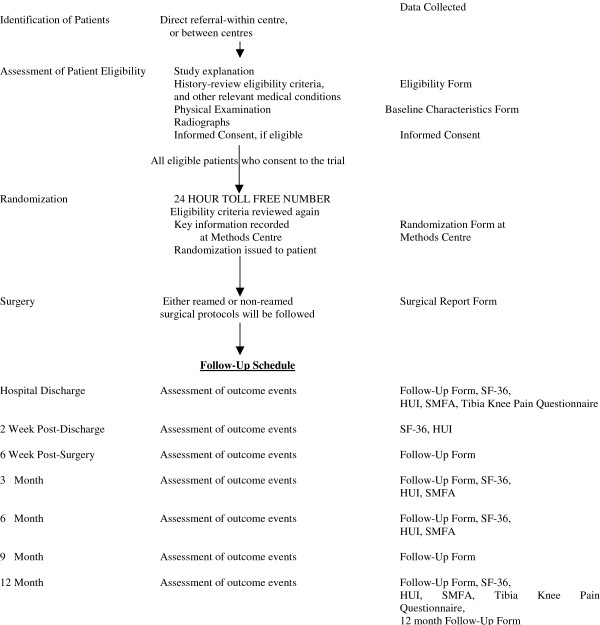
Recruitment and Follow-Up Schedule.

Each randomization package contained the following case report forms: eligible included patient form, baseline characteristics form, medications form (which will collect information on NSAIDS, anti-inflammatory drugs, anti-convulsants, oral steroids, and statins), fracture characteristics form, surgical report form, peri-operative data form, follow up report form (for each visit), Short Musculoskeletal Function Assessment questionnaire, Health Utilities Index questionnaire, Short Form-36 questionnaire, Tibia Knee Pain questionnaire, protocol deviation form, missed follow up form, early withdrawal form, and excluded patient form. Randomization packages containing all the relevant forms were mailed to each center prior to the study start. The research coordinator confirmed receipt and completeness of randomization packages prior to study start. The research coordinator at each participating centre also ensured that all relevant case report forms were complete and faxed to the Methods Centre as soon as completed. Figure 1 summarizes key stages in this process.

Early after we initiated screening and recruitment across participating sites, we identified a higher than expected crossover rate from the non-reamed to the reamed nails. One explanation for crossovers was the apparent enrolment of patients with tibial intramedullary canals that were too narrow to allow the passage of a smaller diameter non-reamed nail. To address this issue, we revised our randomization system to ensure that investigators confirmed at least a 9.5 mm diameter of the tibial intramedullary canal at the isthmus (i.e. the narrowest point) prior to randomizing the patient. This modification assured that patients were eligible for either surgical approach.

### Protecting against sources of bias

#### Adjudication of events

A blinded Central Adjudication Committee (CAC) reviewed reports of all re-operations as soon as possible after each event was reported to the methods center, decided if a re-operation meeting study criteria had occurred, and if so, categorized the nature of the re-operation.

All centres emailed digital photographs of the required x-rays to the S.P.R.I.N.T. methods centre. In addition, site coordinators mailed all relevant hospital records. All relevant blinded patient records (DataFax Case Report Forms, chart notes, and x-rays) were posted on a specially designed, and password protected, internet website for adjudication. We were concerned that the size of the nail would be sufficient to unmask the allocation of treatment as non-reamed nails are smaller in diameter. To mask the allocation of treatment, we photo-edited to crop the digital radiograph to include only the fracture site.

For those fractures that surgeons reported to have less than 50% cortical contact between fracture ends, all adjudicators determined the fracture gap. For all suspected study events, adjudicators judged the following: size of fracture gap, re-operation planned or unplanned, appropriateness of re-operation, and whether it was a study event. Any disagreements were resolved by conference call. If adjudicators could not reach consensus, additional information was requested from the participating site to clarify areas of uncertainty. All decisions made by the committee were final.

### Contamination and co-intervention

Crossovers were likely to occur only when, in the operating room, the surgeon found an extremely narrow intramedullary canal in a patient allocated to non-reamed nailing and undertook limited reaming to allow insertion of a nail. Any patients who did cross were analyzed in the group to which they were allocated, maintaining the intention to treat approach we planned for the analysis.

Surgical co-intervention, such as a general, neurosurgical or orthopaedic procedure, were likely to confound outcomes. Our standardization of management protocols limited co-intervention, and we documented the use of drugs that affected the bone, and major additional procedures that patients underwent (Table 2).

**Table 2:** Major additional procedures in S.P.R.I.N.T patients

	Total (%)	Reamed (%)	Non-Reamed(%)
Additional Procedures (n,%)†			
Yes	310 (25.3)	166 (26.7)	144 (23.8)
Fixation of other LE fracture	132 (42.6)	74 (44.6)	58 (40.3)
Fixation of UE fracture	65 (21.0)	30 (18.1)	35 (24.3)
Fixation of pelvic/acetabular	17 (5.5)	11 (6.6)	6 (4.2)
Upper extremity amputation	0 (0.0)	0 (0.0)	0 (0.0)
Fixation of non-eligible tibial f	27 (8.7)	12 (7.2)	15 (10.4)
Laparotomy	8 (2.6)	3 (1.8)	5 (3.5)
Lower extremity amputation	2 (0.6)	1 (0.6)	1 (0.7)
Craniotomy	4 (1.3)	4 (2.4)	0 (0.0)
Spine fracture	4 (1.3)	3 (1.8)	1 (0.7)
Removal hardware	7 (2.3)	3 (1.8)	4 (2.8)
Other wound closure	91 (29.4)	53 (31.9)	38 (26.4)
Fasciotomy	8 (2.6)	5 (3.0)	3 (2.1)
Fixation of facial fracture	2 (0.6)	1 (0.6)	1 (0.7)
Drain insertion	1 (0.3)	0 (0.0)	1 (0.7)
Knee dislocation repair	2 (0.6)	0 (0.0)	2 (1.4)
(given as % of those with additional fractures)			
No	916 (74.7)	456 (73.3)	460 (76.2)

### Study eligibility criteria

Inclusion criteria.**1**. Men or women who were skeletally mature (age 18 years or older) **2**. Fracture of the tibia that had complete anterioposterior and lateral radiographs **3**. Closed or open fractures (Tscherne Grade 0–3, Gustillo Grade I-IIIB) [57-59]**4**. Fracture that required operative treatment (failure to maintain alignment with conservative treatment). **5**. Provision of informed consent.

Individuals were excluded from the study if they had the following characteristics:

**1**. Fractures were not amenable to intramedullary nailing (<5 cm distal to the tibial tubercle, or < 5 cm proximal to the tibiotalar joint). **2**. Inability to pass an unreamed nail. **3**. Fractures that had associated neurovascular deficits (Gustillo Grade IIIC injuries) [58,59]. **4**. Pathologic fractures. **5**. Surgical delay of > 12 hours from time of injury (open fractures) [60]. **6**. Surgical delay of > 3 weeks from time of injury (closed fractures). **7**. Retained hardware in the affected limb that interfered with intramedullary nailing. **8**. Associated fractures of the foot, ankle, or knee. **9**. Likely problems, in the judgment of the investigators, with maintaining follow-up. We excluded, for example, patients with no fixed address, those who reported a plan to move out of town in the next year, or intellectually challenged patients without adequate family support.

Participating centers identified patients through direct referral. Each surgeon or designated fellow or resident conducted a history and physical examination and completed a check list based upon study eligibility. The resident/fellow or research coordinator obtained informed consent. Figure 1 outlines the key aspects of the recruitment.

We registered all patients who met the inclusion criteria and documented reasons for failure to randomize. The CAC adjudicated all situations where eligibility was in doubt. The CAC included the Study Biostatistician (SW), the Principal Investigator (MB, orthopaedic surgeon), the co-principal investigator (GHG, internist/methodologist), and four orthopaedic surgeons (PT, MFS, DS, EHS).

After the first interim analysis (N = 332 patients), we expanded the eligibility criteria to allow surgeons to use their own best judgment to determine if the fracture was amenable to intramedullary nailing. Specifically, surgeons did not exclude patients whose fractures were <5 cm distal to the tibial tubercle, or <5 cm proximal to the tibiotalar joint if they judged it as amenable to intramedullary nailing. We also revised our exclusion criteria #8 above to the following: Surgeons could randomize a patient if the fractures did not interfere with the intramedullary nailing. We also allowed fractures that could be separately fixed without compromising placement of an intramedullary nail.

### Frequency and duration of follow-up

Follow up continued for a period of one year. At the one-year follow up visit, the surgeon also documented any re-operations that were planned for the patient after one-year. Clinical and radiographic assessments occurred at the time of admission to hospital (baseline), hospital discharge, 2 weeks post discharge, 6 weeks, 3, 6, 9, and 12 months post-intramedullary nailing (Figure 1). Follow up assessment included subsequent operative procedures, the reason for the procedure, and functional status.

Two factors dictated the choice of 1 year follow up period: 1) All participating surgeons agreed that the decision to re-operate on a tibial fracture would occur within the first year following surgery in all, or virtually all, patients. 2) Previous randomized trials had reported closed tibial fractures to heal at a mean 3.9 months [48] and open tibial fractures to heal at a mean 7 months [44]. Thus, all of the closed fracture re-operations, and over 95% of the open fracture re-operations, occur within one year. Since increasing follow up beyond one year would yield very little additional information on re-operation rates, efficient use of resources dictated a one-year follow up.

### Outcome measures

#### Primary outcome

We originally described our primary outcome as re-operation within 1 year of the initial surgery. Surgical procedures considered as primary events included: 1) bone grafts, 2) implant exchange or removal (broken nail or deep infection), and 3) debridement of bone and soft tissue from deep infection. We assessed re-operation at follow up visits of hospital discharge, 6 weeks, 3 months, 6 months, 9 months, and 1 year. The attending physicians documented any subsequent operative procedure directed specifically towards improving fracture healing at each follow up visit.

After the first interim analysis in January 2003, when 332 patients had been enrolled, the event rate was substantially lower than anticipated from our review of previous studies. In response, we proposed, and both the Data Safety and Monitoring Committee (DSMB) (National Institutes of Arthritis, Musculoskeletal and Skin diseases, NIAMS) and the primary funding agency (CIHR) accepted, the adoption of an expanded primary composite outcome. The new primary outcome included re-operation within 1 year of initial surgery in patients with cortical continuity, or less than 1 cm gap between the fracture ends post intramedullary nailing (Table 3). Specific eligible procedures in the composite endpoint included the following: 1) nail replacement, 2) operation for infection, 3) dynamization, 4) removal of locking screw(s) due to hardware breakage (autodynamization) or loosening of screws, 5) drainage of hematomas, and 6) fasciotomy for intra-operative or post-operative compartment syndrome.

**Table 3:** Fracture gaps, protocol deviation status, and study event status

Size of Fracture Gap at the Time of the Initial Surgery*	Protocol Deviation if Re-Operation before 6 Months**	Study Event
0 (no fracture gap)	Yes	Yes – Primary Event
Fracture gap less than 1 cm	Yes	Yes – Primary Event
Fracture gap greater than or equal to 1 cm	No	No

* This refers to the magnitude of circumferential bone loss as judged by the adjudicator on review of the post-operative radiograph, or non-circumferential bone loss as judged by the adjudicator in the presence of cortical continuity of 0 or 25% as judged by the surgeon at the time of operation and recorded on our study forms.

** For nonunion, malunion, dynamization, not infection

Eligible events: To limit a potential bias against the non-reamed nail group among participating surgeons, we proscribed re-operation within the first 6 months following surgery. This allowed sufficient time for most fractures to heal. The only exceptions were subsequent soft tissue coverage procedures, debridement if infection occurred, and nail replacement if nail breakage occurred. Dynamization was allowed prior to 6 months only if a fracture gap remained following insertion of the intramedullary nail. To identify any deviations in protocol, re-operations were noted at the earlier follow up periods (hospital discharge, 6 weeks, and 3 months).

We did not consider the following procedures as study events: 1) re-operations planned at the time of initial surgery; 2) re-operations or dynamizations in patients with a fracture gap of greater than or equal to 1 cm; 3) removal of proximal or distal locking screws after six months that did not dynamize the fracture; 4) soft tissue coverage in the absence of infection; 5) in open fractures, copious irrigation and debridement of soft tissues and contaminated bone repeated as necessary following the initial nailing; 6) amputations; 7) treatment of wound or tissue necrosis in the absence of infection; and 8) re-operations to correct an unacceptable degree of mal-alignment following the initial nailing.

Criteria for undertaking a bone graft included: 1) a fracture with a ≥ 1 cm fracture gap and at least 50% circumferential bone loss at the fracture site and 2) failure of progression of fracture healing for at least 2 months accompanied by clinical symptoms of delayed/nonunion (pain, difficulty weight bearing). Criteria to permit exchange intramedullary nailing included: 1) a <1 cm fracture gap and at least 50% circumferential bone loss [61] and 2) failure of progression of the fracture to heal for at least 2 months accompanied by clinical symptoms of delayed/nonunion (pain, difficulty weight bearing).

We classified the reasons for re-operation as follows: nonunion, malunion (>5 degrees varus/valgus, >10 malrotation degrees, and >1 cm shortening) [62], compartment syndrome, and infection. Criteria for the diagnosis of nonunion included a failure of the fracture to progress towards healing for at least 2 months (although no secondary procedures were allowed for 6 months following the initial intramedullary nailing). This was evident as a persistent fracture line on radiograph and pain on palpation at the fracture site, or the inability to weight bear without pain. We have previously characterized the reliability of utilizing cortical continuity and fracture lines in the assessment of fracture healing [72].

#### Secondary outcome

We measured functional status as a secondary outcome. Patients completed self-administered functional outcome questionnaires under research coordinator supervision at hospital discharge, 2 weeks post discharge, 6 weeks, and 3, 6, 9, and 12 months post-surgery. Questionnaires included a generic health status measurement instrument, the SF-36 [63-65], a generic utility measure (the Health Utilities Index Mark II/III) [66-68], a disease-specific functional measure targeted to patients with lower extremity fractures (Short Musculoskeletal Function Assessment measure, SMFA) [69,70] and a tibial knee pain questionnaire.

The SF-36 questionnaire was developed from the Medical Outcomes Study [63,64]. It is a self-administered, 36 item questionnaire that measures health-related quality of life in eight domains:1) physical functioning, by measuring the ability to perform a variety of daily activities and tasks that require physical effort (10 items); 2) role limitations due to physical problems(4 items); 3) role limitations due to emotional problems (3 items); 4) vitality, measuring perceived level of energy and fatigue (4 items); 5) freedom from bodily pain (2 items); 6) social functioning (2 items); 7) mental health, by measuring both negative and positive emotional states (5 items); and 8) general health perceptions (6 items). The instrument has been extensively validated and has demonstrated good construct validity, high internal consistency, and high test- retest reliability [63,64]. We considered an important difference to correspond to a moderate effect as defined by Cohen [73] as well as a minimally important difference in the SF-36 as reported by Ware [63]. In both cases, the value is 1/2 the standard deviation, equivalent to a 7 point difference in score. Specifying an alpha (α) level = 0.05, a beta(β) = 0.20 (study power = 80), we required a sample of at least 30 patients to ensure detection of a 1/2 standard deviation improvement.

The Short Musculoskeletal Function Assessment (SMFA) is a 46 item questionnaire that has 6 domains: 1) daily activities, 2) emotional status, 3) arm and hand function, 4) mobility, 5) function index and 6) bothersome index. It has been validated as a measure of patient function [69,70].

As part of a subsequent research protocol for an economic analysis we collected patient utilities utilizing the HUI MarkII/III [66-68].

Additional secondary outcomes included dynamization rates, deep infection rates (bone implant interface), compartment syndrome rates and malunion rates (>5 degrees varus/valgus, >10 malrotation degrees, and >1 cm shortening) [62]. Each of these outcomes will be assessed at follow up visits.

### Statistical issues

#### Sample size

Our choice of sample size was based on the anticipated rate of the primary outcome (re-operation). All statistical hypotheses were two-sided. We chose alpha levels of 0.05 for the primary and 0.01 for the secondary outcomes. We evaluated 7 secondary outcomes, but because they were likely to be correlated the Bonferroni correction would have been excessively conservative we chose not to use this approach. The trial was designed to have a statistical power of 80% for the primary comparison.

Previous studies have reported annual re-operation rates in tibial fracture patients which have ranged from 12–44%, with a weighted pooled risk of 31.6 % (95% confidence intervals: 24.9–39.1%) [43]. After conducting our first blinded interim analysis (N = 332 patients) of patients followed for 1 year, our primary event rate (13%) proved lower than projected from previous RCTs. A number of factors account for our lower rate of re-operation. First, our strict protocol for optimal peri-operative management improved patient care. This improvement in care has, we believe, decreased the frequency of re-operations. Second, our proscription of re-operations for delayed healing prior to six months was a feature not regulated in previous studies [43-54]. Third, we excluded re-operations in patients with fracture gaps ≥ 1 cm because it is extremely implausible that the type of nail affects the success in achieving union in such patients. The intervention may still affect infection rates because of the compromise of blood supply associated with reaming. Thus, including re-operations for failure to heal (but not for infection) in such patients would increase the random error in the comparison of the two types of nailing with respect to our primary outcomes. Prior studies included re-operations for failure to heal in patients with fracture gaps greater than 1 cm. Of our first 74 patients with re-operations, 10 had fracture gaps greater than 1 cm and of these, all had re-operations because of failure of union. With our event rate of 13%, an additional 300 patients (total sample size = 1200 patients) provided adequate study power (87%) for a treatment effect of at least 37%.

We planned *a priori *to separately compare the effects of reamed versus nonreamed intramedullary nailing in patients with closed and with open tibial shaft fractures. We adjusted all analyses for important prognostic factors. Our interim analysis suggested about 30% of our patients had open fractures (101/332). Thus, we anticipated 360 patients (180 per treatment arm) with open fractures at the conclusion of our trial. With an event rate of 20% from the interim analysis of open fractures (N = 332 patients), our study would have 81% power to detect a 55% relative risk reduction (RRR), 72% power for a 50% RRR, 49% power for a 40% RRR and 38% power for a 35% RRR in re-operation risk with reamed nails. If our point estimate from our previous meta-analysis was correct (RRR = 56%) [43], we would have adequate study power for our analysis.

#### Analysis

Our primary analysis followed the intention-to-treat principle and compared the proportion of patients with a re-operation in the reamed group to the proportion of patients with a re-operation in the non-reamed group at 1 year follow up using a Mantel-Haenszel stratified analysis. We stratified by center and whether the fracture was open or closed. The results of the primary analysis were presented as a relative risk. In addition to the analysis of our primary composite outcome, we also compared the occurrence of each component of the composite in the two groups.

Additional analyses, employing log binomial regression, examined and controlled for the influence of patient and surgical factors that were hypothesized *a priori *to be associated with the risk of re-operation, including age, smoking history, diabetes, fracture location (Proximal, Middle, Distal), fracture comminution (Orthopaedic Trauma Association fracture type), fracture grade (Gustilo or Tscherne grading) and unilateral versus bilateral fractures.

Subgroup analyses were conducted using tests for interactions; all were specified *a priori*. Our subgroup analysis of primary interest was open versus closed fractures. Additional subgroup hypotheses included impact of treatment in multi-trauma versus isolated fractures; unilateral versus bilateral tibial fractures; OTA classification C types vs. B and A; surgeries performed by surgeons versus fellows and residents; and fracture gaps greater than versus less than, or equal to, 1 cm. Within open fractures, we considered subgroups of Types IIIA and IIIB vs. Types I-II. Within closed fractures, we considered subgroups of Tscherne Types 2–3 vs. 0–1 fractures.

Blinded results were initially presented to the Steering Committee members as treatment A and treatment B. Prior to unblinding themselves, Steering Committee members developed two alternative interpretations of the results if A were the 'reamed group' or if A were the 'unreamed' group.

#### Frequency of analyses

We conducted one interim analysis after 322 patients had completed the trial. The data analyst presented the results of these analyses, including confidence intervals, to an independent Data Safety and Monitoring Committee (DSMB). DSMB members were guided by the O'Brien-Fleming stopping rule. In choosing the significance level for the interim analysis to maintain the overall specified type I error rate of 0.05. In the interim analysis, we set the significance level at 0.0006 thus maintaining a significance level of 0.05 for the final analysis. This method was conservative, making it difficult to stop the trial early unless a large difference between treatments was observed. No one other than committee members were aware of the interim results, and only the DSMB committee members were aware of the content of their deliberations.

#### Compliance

We instituted several strategies to ensure adherence to the protocol which including: 1) weekly quality report forms to flag protocol violations, 2) monthly newsletters re-emphasizing important aspects of the protocol, 3) immediate personal telephone calls from the Principal Investigator, or Project Manager to site investigators when protocol violations occurred, 4) 24 hour pager for any queries regarding the study protocol, and 5) annual investigator meetings to discuss protocol violations and strategies to improve adherence. These strategies led to adherence rates of over 90% in important aspects of the study protocol among the first 332 patients included.

Given the inherent variability in practice patterns among orthopaedic surgeons, it was important to ensure that surgeons adhered as closely as possible to the surgical management protocol. In general, large studies involving multiple centres are more feasible if they require minimal changes from current practice. To limit bias that could occur if participating surgeons have different re-operation criteria for reamed and non-reamed nail groups, we proscribed re-operation within the first 6 months following surgery.

Crossovers that occur at the time of surgery are another important protocol violation. In our first 620 patients enrolled, we identified a total of 33 (5.3%) crossovers across both surgical interventions. In response to the higher than expected crossover rate, the Steering Committee reinforced the importance of adherence to protocol via: 1) direct telephone calls from the Principal Investigator to each site at which a crossover had occurred, 2) a conference call of all site investigators to highlight this finding, and 3) a focused discussion of protocol violation at our annual investigators' meeting.

#### Loss to follow-up

Previous randomized trials in orthopaedic surgery have typically reported 10% but up to 30% loss to follow up of included patients [43-52]. To avoid this problem and achieve high follow-up rate we successfully implemented the following procedures: 1) We excluded individuals who were likely to be difficult with follow-up (see exclusion criteria). 2) At the time of randomization, each patient provided their own address and phone number, the name and address of their primary care physician, and the name, address and phone number of three people at different addresses with whom the patient did not live who were likely to be aware of the patient's whereabouts. The research coordinator confirmed that these numbers were accurate prior to the patient's discharge from hospital. 3) Participants received information on tibial fractures, their complications and the potential treatment effects, expectations for personal benefit from study participation, and motivation for adherence with follow up visits and research protocols. Aids used for education and support included a patient information booklet, a telephone number for advice in case of complications or questions regarding follow up visits. 4) Patients received reminders for upcoming clinic visits from local study personnel. 5) Follow up schedules coincided with normal surgical fracture clinic visits. 6) Study personnel contacted patients at least once every three months to obtain information about any planned change in residence. 7) If a patient refused to return for a follow up assessment, his/her status with regard to re-operation or any secondary outcome was determined by telephone contact with the patient, alternate contact, or the family physician.

#### Committees and participating centres

**Study Trial Co-Principal Investigators: **Mohit Bhandari; Gordon Guyatt; **Steering Committee: **Gordon Guyatt (chair); Mohit Bhandari; David W. Sanders; Emil H. Schemitsch; Marc Swiontkowski; Paul Tornetta III; Stephen Walter; **Central Adjudication Committee: **Gordon Guyatt (chair); Mohit Bhandari; David W. Sanders; Emil H. Schemitsch; Marc Swiontkowski.; Paul Tornetta III; Stephen Walter; **SPRINT Methods Centre Staff: McMaster University, Hamilton, Ontario: **Sheila Sprague; Diane Heels-Ansdell; Lisa Buckingham; Pamela Leece; Helena Viveiros; Tashay Mignott; Natalie Ansell; Natalie Sidorkewicz; **University of Minnesota, Minneapolis, Minnesota: Julie Agel; Data Safety and Monitoring Board (DSMB): **Claire Bombardier (chair); Jesse A. Berlin; Michael Bosse; Bruce Browner; Brenda W. Gillespie; Peter O'Brien; **Site Audit Committee: **Julie Agel; Sheila Sprague; Rudolf Poolman; Mohit Bhandari. **Writing Committee: **Mohit Bhandari (chair); Gordon Guyatt; David W. Sanders; Emil H. Schemitsch; Marc Swiontkowski; Paul Tornetta III; Stephen Walter

**Investigators: London Health Sciences Centre/University of Western Ontario, London, Ontario: **David W. Sanders; Mark D. Macleod; Timothy Carey; Kellie Leitch; Stuart Bailey; Kevin Gurr; Ken Konito; Charlene Bartha; Isolina Low; Leila V. MacBean; Mala Ramu; Susan Reiber; Ruth Strapp; Christina Tieszer; **Sunnybrook Health Sciences Centre/University of Toronto, Toronto, Ontario: **Hans Kreder; David J. G. Stephen; Terry S. Axelrod; Albert J.M. Yee; Robin R. Richards; Joel Finkelstein; Richard M. Holtby; Hugh Cameron; John Cameron; Wade Gofton; John Murnaghan; Joseph Schatztker; Beverly Bulmer; Lisa Conlan; **Hospital du Sacre Coeur de Montreal, Montreal, Quebec: **Yves Laflamme; Gregory Berry; Pierre Beaumont; Pierre Ranger; Georges-Henri Laflamme; Alain Jodoin; Eric Renaud; Sylvain Gagnon; Gilles Maurais; Michel Malo; Julio Fernandes; Kim Latendresse; Marie-France Poirier; Gina Daigneault; **St. Michael's Hospital/University of Toronto, Toronto, Ontario: **Emil H. Schemitsch; Michael M. McKee; James P. Waddell; Earl R. Bogoch; Timothy R. Daniels; Robert R. McBroom; Robin R. Richards; Milena R. Vicente; Wendy Storey; Lisa M. Wild; **Royal Columbian Hospital/University of British Columbia, Vancouver, British Columbia: **Robert McCormack; Bertrand Perey; Thomas J. Goetz; Graham Pate; Murray J. Penner; Kostas Panagiotopoulos; Shafique Pirani; Ian G. Dommisse; Richard L. Loomer; Trevor Stone; Karyn Moon; Mauri Zomar; **Wake Forest Medical Center/Wake Forest University Health Sciences, Winston-Salem, North Carolina: **Lawrence X. Webb; Robert D. Teasdall; John Peter Birkedal; David Franklin Martin; David S. Ruch; Douglas J. Kilgus; David C. Pollock; Mitchel Brion Harris; Ethan Ron Wiesler; William G. Ward; Jeffrey Scott Shilt; Andrew L. Koman; Gary G. Poehling; Brenda Kulp; **Boston Medical Center/Boston University School of Medicine, Boston, Massachusetts: **Paul Tornetta III; William R. Creevy; Andrew B. Stein; Christopher T. Bono; Thomas A. Einhorn; T. Desmond Brown; Donna Pacicca; John B. Sledge III; Timothy E. Foster; Ilva Voloshin; Jill Bolton; Hope Carlisle; Lisa Shaughnessy; **Wake Medical Center, Raleigh, North Carolina: **William T. Ombremsky; C. Michael LeCroy; Eric G. Meinberg; Terry M. Messer; William L. Craig III; Douglas R. Dirschl; Robert Caudle; Tim Harris; Kurt Elhert; William Hage; Robert Jones; Luis Piedrahita; Paul O. Schricker; Robin Driver; Jean Godwin; Gloria Hansley; **Vanderbilt University Medical Center, Nashville, Tennessee: **William Todd Obremskey; Philip James Kregor; Gregory Tennent; Lisa M. Truchan; Marcus Sciadini; Franklin D. Shuler; Robin E. Driver; Mary Alice Nading; Jacky Neiderstadt; Alexander R. Vap; **MetroHealth Medical Center, Cleveland, Ohio: **Heather A. Vallier; Brendan M. Patterson; John H. Wilber; Roger G. Wilber; John K. Sontich; Timothy Alan Moore; Drew Brady; Daniel R. Cooperman; John A. Davis; Beth Ann Cureton; **Hamilton Health Sciences, Hamilton, Ontario: **Scott Mandel; R. Douglas Orr; John T.S. Sadler; Tousief Hussain; Krishan Rajaratnam; Bradley Petrisor; Mohit Bhandari; Brian Drew; Drew A. Bednar; Desmond C.H. Kwok; Shirley Pettit; Jill Hancock; Natalie Sidorkewicz; **Regions Hospital, Saint Paul, Minnesota: **Peter A. Cole; Joel J. Smith; Gregory A. Brown; Thomas A. Lange; John G. Stark; Bruce Levy; Marc F. Swiontkowski; Julie Agel; Mary J. Garaghty; Joshua G. Salzman; Carol A. Schutte; Linda (Toddie) Tastad; Sandy Vang; **University of Louisville School of Medicine, Louisville, Kentucky: **David Seligson; Craig S. Roberts; Arthur L. Malkani; Laura Sanders; Sharon Allen Gregory; Carmen Dyer; Jessica Heinsen; Langan Smith; Sudhakar Madanagopal; **Memorial Hermann Hospital, Houston, Texas: **Kevin J. Coupe; Jeffrey J. Tucker; Allen R. Criswell; Rosemary Buckle; Alan Jeffrey Rechter; Dhiren Shaskikant Sheth; Brad Urquart; Thea Trotscher; **Erie County Medical Center/University of Buffalo, Buffalo, New York: **Mark J. Anders; Joseph M. Kowalski; Marc S. Fineberg; Lawrence B. Bone; Matthew J. Phillips; Bernard Rohrbacher; Philip Stegemann; William M. Mihalko; Cathy Buyea; **University of Florida – Jacksonville, Jacksonville, Florida: **Stephen J. Augustine; William Thomas Jackson; Gregory Solis; Sunday U. Ero; Daniel N. Segina; Hudson B. Berrey; Samuel G. Agnew; Michael Fitzpatrick; Lakina C. Campbell; Lynn Derting; June McAdams; **Academic Medical Center, Amsterdam, The Netherlands: **J. Carel Goslings; Kees Jan Ponsen; Jan Luitse; Peter Kloen; Pieter Joosse; Jasper Winkelhagen; Raphaël Duivenvoorden; **University of Oklahoma Health Science Center, Oklahoma City, Oklahoma: **David C. Teague; Joseph Davey; J. Andy Sullivan; William J. J. Ertl; Timothy A. Puckett; Charles B. Pasque; John F. Tompkins II; Curtis R. Gruel; Paul Kammerlocher; Thomas P. Lehman; William R. Puffinbarger; Kathy L. Carl; **University of Alberta/University of Alberta Hospital, Edmonton, Alberta: **Donald W. Weber; Nadr M. Jomha; Gordon R. Goplen; Edward Masson; Lauren A. Beaupre; Karen E. Greaves; Lori N. Schaump; **Greenville Hospital System, Greenville, South Carolina: **Kyle J. Jeray; David R. Goetz; Davd E. Westberry; J. Scott Broderick; Bryan S. Moon; Stephanie L. Tanner; **Foothills General Hospital, Calgary, Alberta: **James N. Powell; Richard E. Buckley; Leslie Elves; **Saint John Regional Hospital, Saint John, New Brunswick: **Stephen Connolly; Edward P. Abraham; Donna Eastwood; Trudy Steele; **Oregon Health & Sciences University, Portland, Oregon: **Thomas Ellis; Alex Herzberg; George A. Brown; Dennis E. Crawford; Robert Hart; James Hayden; Robert M. Orfaly; Theodore Vigland; Maharani Vivekaraj; Gina L. Bundy; **San Francisco General Hospital, San Francisco, California: **Theodore Miclau III; Amir Matityahu; R. Richard Coughlin; Utku Kandemir; R. Trigg McClellan; Cindy Hsin-Hua Lin; **Detroit Receiving Hospital, Detroit, Michigan: **David Karges; Kathryn Cramer; J. Tracy Watson; Berton Moed; Barbara Scott; **Deaconess Hospital Regional Trauma Center and Orthopaedic Associates, Evansville, Indiana: **Dennis J. Beck; Carolyn Orth; **Thunder Bay Regional Health Science Centre, Thunder Bay, Ontario: **David Puskas; Russell Clark; Jennifer Jones; **Jamaica Hospital, Jamaica, New York: **Kenneth A. Egol; Nader Paksima; Monet France; **Ottawa Hospital – Civic Campus, Ottawa, Ontario: **Eugene K. Wai; Garth Johnson; Ross Wilkinson; Adam T. Gruszczynski; Liisa Vexler.

## Discussion

Trials in orthopaedic surgery have typically been single center initiatives that often lack sufficient power to make recommendations regarding surgical practice, and suffer from methodological limitations. This is the first large scale co-operative trial among orthopaedic traumatologists in North America and Europe to assess an orthopaedic surgical treatment. Our team of investigators will be able to work towards answering many other important questions in orthopaedic surgery. This trial will also establish a model for multi-center co-operative trials in orthopaedic trauma, which, over the long term, will have the same sort of impact as large trials in cardiovascular medicine and cerebrovascular surgery.

S.P.R.I.N.T has important strengths including a large sample of enrolled patients, multiple participating surgeons and centers, a patient-important outcome with independent adjudication and improved methodological rigor in the conduct of the trial (including a proscription to re-operation before 6 months).

A major strength of our study was the assurance of allocation concealment with remote 24 hour randomization. The extent to which previous smaller randomized trials maintained concealment remains questionable. Lack of allocation concealment in trials has been shown to over-estimate treatment effects. S.P.R.I.N.T's sample size is ten-fold greater than the largest previous randomized trial evaluating reamed and non-reamed tibial nails. Based on previous trials and our beliefs that treatment effects would be conserved across open and closed fractures, S.P.R.I.N.T is powered to detect patient-important differences in re-operation risk.

One major methodological safeguard employed in S.P.R.I.N.T but not in previous studies was the 6 month proscription of re-operation. Previous trials with large reported benefits to reamed nails and S.P.R.I.N.T surgeons' increased experiences with reamed nails risked a differential threshold towards early re-operation in patients randomized to non-reamed nail insertion. Our choice of 6 months was based on a consensus of participating surgeons that, if left alone, 6 months was adequate to allow most treated fractures to heal in either group.

One major limitation of S.P.R.I.N.T was the fact that surgeons could not be blinded to treatment allocation leaving the assessment of outcomes and decisions to re-operate vulnerable to bias. To limit such bias, we used an objective primary outcome and independently adjudicated all primary outcome events by a committee of 7 members, of whom 5 were orthopaedic trauma surgeons. The primary composite outcome of re-operation provides a patient-important estimate of effect superior to previously described measures such as radiographic fracture healing, delayed unions, and nonunions.

S.P.R.I.N.T, as any trial evaluating two procedures requiring technical experience, risked differential expertise bias [72]. In a survey of 139 S.P.R.I.N.T surgeons (response rate: 74, 52%), 55% had used the reamed nailing technique in at least 20 patients in the year preceding the trial whereas only 19% used non-reamed nail insertion. The median number of cases surgeons had performed in the year before participation in S.P.R.I.N.T. was 12 reamed procedures and 2 non-reamed procedures (median difference 7 procedures, 95% confidence interval 5 to 11). The extent to which a differential expertise bias was operating in S.P.R.I.N.T remains unknown.

The treatment of tibial fractures remains controversial. Identifying treatment alternatives that reduce the risk of a subsequent operation as well as costs to the health care system will be a significant contribution to the practice of orthopaedics. This trial will not only change current orthopaedic practice, but will set a benchmark for the conduct of future orthopaedic trials.

## Competing interests

The author declares that they have no competing interests.

## Authors' contributions

The Writing Committee [Mohit Bhandari MD (Chair), Gordon Guyatt MD, Paul Tornetta III MD, Emil Schemitsch MD, Marc Swiontkowski MD, David Sanders MD, and Stephen D. Walter, PhD] assumes responsibility for the overall content and integrity of the manuscript. MB and GG, as Principal Investigators, had full access to the study data and take responsibility for its integrity and the accuracy of the analyses. MB, GG, SW, PT III, ES, DS, MS contributed to the study concept and design. MB and GG drafted the manuscript. MB, GG, SW, PT III, ES, DS, and MS are responsible for the critical revision of the manuscript for important intellectual content. MB, GG, MS, PT III obtained funding for this trial. MB, GG, SW, PT III, ES, DS, MS supervised the study. All authors read and approved the final manuscript.

## Pre-publication history

The pre-publication history for this paper can be accessed here:


